# NLRP6 Serves as a Negative Regulator of Neutrophil Recruitment and Function During *Streptococcus pneumoniae* Infection

**DOI:** 10.3389/fmicb.2022.898559

**Published:** 2022-05-25

**Authors:** Qi Tao, Dongyi Xu, Kaixiang Jia, Xinrui Cao, Chao Ye, Sanlei Xie, Dong-Liang Hu, Lianci Peng, Rendong Fang

**Affiliations:** ^1^Joint International Research Laboratory of Animal Health and Animal Food Safety, College of Veterinary Medicine, Southwest University, Chongqing, China; ^2^Department of Medical Microbiology and Infection Prevention at University of Groningen/University Medical Center Groningen, Groningen, Netherlands; ^3^Department of Zoonoses, Kitasato University School of Veterinary Medicine, Towada, Japan; ^4^Chongqing Key Laboratory of Herbivore Science, Chongqing, China

**Keywords:** *Streptococcus pneumoniae*, NLRP6, neutrophils, neutrophil extracellular traps, host defense

## Abstract

*Streptococcus pneumoniae* is an invasive pathogen with high morbidity and mortality in the immunocompromised children and elderly. NOD-like receptor family pyrin domain containing 6 (NLRP6) plays an important role in the host innate immune response against pathogen infections. Our previous studies have shown that NLRP6 plays a negative regulatory role in host defense against *S. pneumoniae*, but the underlying mechanism is still unclear. The further negative regulatory role of NLRP6 in the host was investigated in this study. Our results showed that NLRP6^−/−^ mice in the lung had lower bacterial burdens after *S. pneumoniae* infection and expressed higher level of tight junction (TJ) protein occludin compared to WT mice, indicating the detrimental role of NLRP6 in the host defense against *S. pneumoniae* infection. Transcriptome analysis showed that genes related to leukocytes migration and recruitment were differentially expressed between wild-type (WT) and NLRP6 knockout (NLRP6^−/−^) mice during *S. pneumoniae* infection. Also, NLRP6^−/−^ mice showed higher expression of chemokines including C-X-C motif chemokine ligand 1 (CXCL1) and 2 (CXCL2) and lower gene expression of complement C3a receptor 1 (C3aR1) and P-selectin glycoprotein ligand-1 (PSGL-1) which are the factors that inhibit the recruitment of neutrophils. Furthermore, NLRP6^−/−^ neutrophils showed increased intracellular bactericidal ability and the formation of neutrophil extracellular traps (NETs) during *S. pneumoniae* infection. Taken together, our study suggests that NLRP6 is a negative regulator of neutrophil recruitment and function during *S. pneumoniae* infection. Our study provides a new insight to develop novel strategies to treat invasive pneumococcal infection.

## Introduction

*Streptococcus pneumoniae* is a major pathogen of human, causing severe diseases such as sinusitis, otitis media, meningitis, and pneumonia associated with high morbidity and mortality (Weiser et al., 2018). *S. pneumoniae* primarily infects immunocompromised elderly and children, which cause hundreds of thousand deaths of children each year in the world (O’Brien et al., 2009; Engholm et al., 2017). Nowadays, *S. pneumoniae* has been listed as one of 12 high priority pathogens by the World Health Organization (Brazel et al., 2022). In addition, the emergence of antibiotics-resistant bacteria brings a great challenge to treat infections.

*S. pneumoniae* infection firstly occurs in the respiratory tract where they recruit neutrophils and macrophages to kill bacteria (Standish and Weiser, 2009). Neutrophils considered as the quickest responder immune cell play an important role in the host for the clearance of bacteria (Kolaczkowska and Kubes, 2013). They kill invading pathogens through phagocytosis, degranulation, and release of NETs (Capucetti et al., 2020). Neutrophil recruitment is a complex process which is regulated by multiple factors including chemokines and some host signaling events (Zhang et al., 2010; Paudel et al., 2019a). Recently, it has been reported that NLRP6 inflammasome regulates the recruitment of neutrophils during *Listeria monocytogenes* (*L. monocytogenes*), *Staphylococcus aureus* (*S. aureus*), and *Klebsiella pneumoniae* infections (Anand et al., 2012; Ghimire et al., 2018; Cai et al., 2021). However, whether NLRP6 inflammasome is involved in the modulation of recruitment of neutrophils during *S. pneumoniae* is still unknown.

NLRP6 belongs to the family of nucleotide binding oligomerization domain-like receptors (NLRs) proteins and its role in the host defense has been extensively studied (Zheng et al., 2021). NLRP6 exhibits diametrically opposite functions for different pathogenic infections and even in different organs or cells (Ghimire et al., 2020). It has been reported that NLRP6 is highly expressed in the intestine and maintains intestinal homeostasis by regulating the secretion of mucus from goblet cells to protect the host against *Citrobacter rodentium* infection (Wlodarska et al., 2014). NLRP6 also exerts an antiviral effect in host intestinal epithelial cells by enhancing the massive expression of type I/III interferons (Wang et al., 2015). Furthermore, NLRP6 regulates neutrophil homeostasis and protects the host against *K. pneumoniae* (Cai et al., 2021). On the other hand, NLRP6 has been proven to negatively regulate the immune response in the host. For example, NLRP6^−/−^ mice were less susceptible to bacteria and exhibited higher survival and bacterial clearance during *L. monocytogenes*, *Salmonella*, *Escherichia coli* and *S. aureus* infection (Anand et al., 2012; Ghimire et al., 2018), indicating the negative role in the host. Similarly, our previous study has shown that NLRP6 plays a detrimental role in the host defense against *S. pneumoniae* infection, and *S. pneumoniae*-infected NLRP6^−/−^ mice displayed increased recruitment of neutrophils and macrophages (Xu et al., 2021). However, the exact mechanism by which NLRP6 negatively regulates host defense against *S. pneumoniae* remains unknown.

In this study, NLRP6^−/−^ mice and peritoneal neutrophils were used to investigate the in-depth mechanism by which NLRP6 plays a negative role during *S. pneumoniae* infection. Our study showed the NLRP6 inhibited the recruitment of macrophages and neutrophils to the lung through decreasing the chemokine expression including CXCL1 and CXCL2 during *S. pneumoniae* infection. Moreover, NLRP6 attenuated neutrophil intracellular bactericidal killing by inhibiting the formation of NETs.

## Materials and Methods

### Bacterial Strains and Growth Conditions

*S. pneumoniae* D39 was kindly provided by Professor Kohsuke Tsuchiya, Kanazawa University. *S. pneumoniae* were cultured on blood agar plates and grown overnight at 37°C with 5% CO_2_. Single colony was grown in Todd-Hewitt broth (Hope Biotech, Qingdao, China) supplemented with 0.5% yeast extract (THY). Bacteria were incubated at 37°C for about 6–8 h until grown to the mid-log phase (OD600 = 0.4–0.6). Finally, the concentration was determined by counting on blood agar plates and bacteria was diluted for being used for assays described below.

### Mice

The C57BL/6 mice were purchased from Chongqing Academy of Chinese Material Medical (Chongqing, PR China). NLRP6^−/−^ mice were kindly provided by Feng Shao from the National Institute of Biological Sciences (Beijing, PR China). All gene knockout mice were on a C57BL/6 background and maintained in Specific Pathogen Free (SPF) conditions for being used at 7–9 weeks old. This study was approved by Institutional Animal Care and Use Committee (IACUC) of Southwest University, Chongqing, China (IACUC-20210215-06).

### Neutrophils

Neutrophil isolation was performed as described previously (Hou et al., 2021). Briefly, mice were intraperitoneally stimulated with 2 ml of 4% thioglycolate medium (Eiken, Tokyo, Japan). After 3–4 h, the mice peritoneal exudate cells (PECs) were obtained by peritoneal lavage and suspended with RPMI 1640 (Gibco, Gaithersburg, United States) supplemented with 10% FCS (fetal calf serum) or Opti-MEM (Gibco, Gaithersburg, United States). Then, the cells were seeded at a density of 2 × 10^5^ cells/well in 48-well plates or at a density of 5 × 10^5^ cells/well in 12-well plates. The purity of neutrophils was detected by flow cytometry. More than 90% of PECs were Gr-1^+^ cells, and F4/80^+^ cells were less than 10%. These cells were maintained at 37°C with 5% CO_2_ before being used for assays described below.

### Macrophages

Mice were intraperitoneally stimulated with 2 ml of 4% thioglycolate medium and PECs were collected 4 days later. Cells were washed by RPMI and suspended in RPMI with 10% FCS. After 2 h incubation, the nonadherent cells were removed and adherent cells were used for assays described below. Flow cytometry showed that more than 95% of adherent PECs were F4/80^+^ cells.

### Transcriptome Analysis

WT and NLRP6^−/−^ mice were infected with D39 at a dose of 5 × 10^7^ CFU in 20 μl PBS by intranasal inoculation. After 24-h infection, mice were euthanized and then lung tissues were collected and quickly frozen in liquid nitrogen. Samples were sent to the Shanghai Personalbio Technology Co., Ltd. for transcriptome sequencing and analysis (HiSeq, Illumina, United States). RNA-sequencing reads were first trimmed to remove poly(A) and unqualified reads with *cutadapt*, then aligned to Mus musculus GRCm38 genome with *Ensembl*, and RNA read depth of 6G. The numbers of counts were summarized at the gene level using *HTseq*. Gene expression values were computed from fragments per kilo bases per million fragments (FPKM) values produced by addition of a pseudocount of 1 and log2 transformation of the results. Paired differential gene expression analyses were performed with *DEseq* R package by addition of fold change >2 and value of *p* <0.05. Volcano plots of these differential genes were drawing with *ggplots2* R package. The terms or pathways involved in host antiviral immune response with value of *p*<0.05 and FDR q-value <0.05 were used to drawing GO term enrichment or KEGG pathway enrichment.

### Real-Time Polymerase Chain Reaction

Total RNA was extracted from infected lung tissues of mice using TRIzol reagent (Tiangen, Beijing, China) according to the manufacturer’s instructions. Then, cDNA was synthesized using PrimeScript RT reagent Kit (TaKaRa, Dalian, China). Subsequently, quantitative real-time PCR was carried out using SsoFast Eva Green Super-Mix (Bio-Rad, Hercules, CA, United States) and performed on a Bio-Rad CFX 96 instrument. All reactions were performed in triplicate and normalized against β*-actin* following the 2^−ΔΔCt^ way. The primers used in this study were designed and synthesized as follows: CXCL1 forward 5′-ACT GCA CCC AAA CCG AAG TC and reverse 5′-TGG GGA CAC CTT TTA GCA TCT T, CXCL2 forward 5′-CCA ACC ACC AGG CTA CAG G and reverse 5′-GCG TCA CAC TCA AGC TCT G, IL-6 forward 5′-CTG CAA GAG ACT TCC ATC CAG and reverse 5′-AGT GGT ATA GAC AGG TCT GTT GG, PSGL-1 5′-CCC TGG CAA CAG CCT TCA G and reverse 5′-GGG TCC TCA AAA TCG TCA TCC, C3aR1 5′-TCT CAG TGT GCT TGA CTG AGC CAT and reverse 5′-AGA CCA AGA ATG ACC ATG GAG GCA, β-actin forward 5′-TGG AAT CCT GTG GCA TCC ATG AAA C and reverse 5′-TAA AAC GCA GCT CAG TAA CAG TCC G.

### Enzyme Linked Immunosorbent Assay

WT and NLRP6^−/−^ mice were infected with D39 as described above. After 24-h infection, mice were euthanized and then lung tissues were collected for protein extraction. The protein expression levels of CXCL1, CXCL2, and IL-6 were determined by ELISA according to the manufacturer’s protocols. The kits of CXCL1 and CXCL2 were purchased from MultiSciences (Hangzhou, China), and IL-6 was purchased from Invitrogen (CA, United States).

### Cell Viability

Neutrophils and macrophages from NLRP6^−/−^ and WT mice were prepared in 48-well plates and infected with D39 for 12 and 24 h, respectively. After infection, 10% WST-reagent was added according to manufacturer’s protocols. Finally, absorbance was measured at 450 nm with a microplate reader (Bio-Rad, Japan) and was corrected for absorbance at 630 nm.

### Cell Intracellular Killing Assay

Neutrophils and macrophages from NLRP6^−/−^ and WT mice were prepared as described above and then were infected with D39 at a multiplicity of infection (MOI) of 10 for 30, 60, and 90 min. After infection at different time points, cells were treated with 100 μg/ml gentamicin for 30 min to kill extracellular bacteria. Next, cells were washed with PBS three times and lysed in PBS containing 0.1% Triton X-100. The lysates were diluted and plated on blood agar plates. After overnight incubation, viable bacteria were quantified.

### Immunofluorescent Staining

NETs were analyzed using immunofluorescent staining as reported previously (Ullah et al., 2017). Neutrophils were seeded on 8-mm sterile glass coverslips at a density of 2 × 10^5^ cells/well with RPMI 1640 containing 10% FCS for 2 h and then were infected with D39 (MOI = 1) for 4 h. After infection, cells were fixed with 4% paraformaldehyde for 30 min. Then, cells were washed with PBS three times, and permeabilized with 0.1% Triton-X 100. After blocking with 5% BSA, cells were incubated with rabbit monoclonal anti-Histone H3 (citrulline R17; Abcam, Cambridge, United Kingdom) for 2 h and goat polyclonal secondary anti-rabbit IgG-H&L Alexa Fluor^®^ 594 (Abcam, Cambridge, United Kingdom) for 1 h. Finally, cells were mounted using Mounting Medium (Solarbio, Beijing, China). DAPI was used to stain cell nuclei. Slides were observed on a fluorescent-inverted microscope (Olympus, Japan).

### Western Blot Analysis

Neutrophils were prepared as described above and then infected with D39 for 4 h. After infection, supernatant and cell lysate were collected. Protein concentrations from lung tissues or cultured neutrophils were measured using BCA protein detection kit (Beyotime, Beijing, China). Then, proteins were separated by 12% sodium dodecyl sulfate-polyacrylamide gel under reducing conditions and transferred to polyvinylidene difluoride (PVDF) membranes. Next, the membranes were blocked with 5% nonfat dry milk and immunoblotted with rabbit monoclonal anti-Histone H3 (citrulline R17), recombinant anti-occludin antibody (Abcam, Cambridge, United Kingdom), streptavidin-horseradish peroxidase (HRP)-conjugated goat anti-rabbit IgG (H + L) antibody (Beyotime, Beijing, China), HRP-conjugated donkey anti-goat IgG(H + L) antibody (Beyotime, Beijing, China). β-actin was employed as a loading control for the cell lysates and detected by mouse anti-β-actin antibody (Beyotime, Beijing, China). Finally, the specific protein bands were detected by ECL detection reagent (Beyotime, Beijing, China).

### Statistical Analysis

Statistical analysis was performed using GraphPad Prism 8.0 software (San Diego, CA). Data are represented as mean ± standard deviation (SD) of three independent (*in vitro*) for each group with triplicate (*n* = 3) or two independent (*in vivo*) experiments for each group with four or five mice. Student′ s t-test was used to analyze the statistical differences for comparisons between two groups. Statistical significance was shown as ^*^p < 0.05, ^**^p < 0.01, and ^***^p < 0.001.

## Results

### NLRP6 Deficiency Reduces Lung Bacterial Burden and Maintains Tight Junction Protein Expression During *Streptococcus pneumoniae* Infection

**Figure 1:**
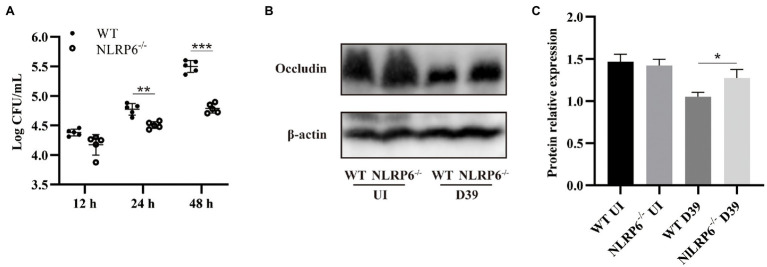
NLRP6 deficiency reduces lung bacterial burden and maintains tight junction protein expression during *S. pneumoniae* infection. WT and NLRP6^−/−^ mice were intranasally infected with *S. pneumoniae* (5 × 10^7^ CFU). **(A)** Lungs were harvested and colony counting was performed at 12 h, 24 h, and 48 h post-infection (*n* = 5/group). **(B)** Lung tissue lysates were collected at 24 h post-infection and were analyzed by western blot to detect expression of occludin (*n* = 5/group). Ratio of occludin levels against β-actin levels was quantified **(C)**. Statistical significance was shown as *p < 0.05, **p < 0.01, and ***p < 0.001.

Our previous study has shown that NLRP6^−/−^ mice have milder inflammatory responses and higher survival rates in *S. pneumoniae* infection (Xu et al., 2021). Similarly, our results showed that WT mice had higher bacterial burdens in the lungs at both 24 and 48 h post-infection compared to NLRP6^−/−^ mice (Figure 1A). It is well-known that tight junction protein function is a sign of intact epithelial barrier. To determine the effect of NLRP6 on TJs during *S. pneumoniae* infection, protein expression levels of occludin in the lungs was examined after 24-h infection. The results showed that the expression level of occludin in NLRP6^−/−^ mice was increased compared with WT mice after *S. pneumoniae* infection (Figures 1B,C). These results indicate NLRP6 deficiency shows the host protection during *S. pneumoniae* infection.

### DEGs Identification of Lung Tissue From *Streptococcus pneumoniae*-Infected WT and NLRP6^−/−^ Mice

**Figure 2:**
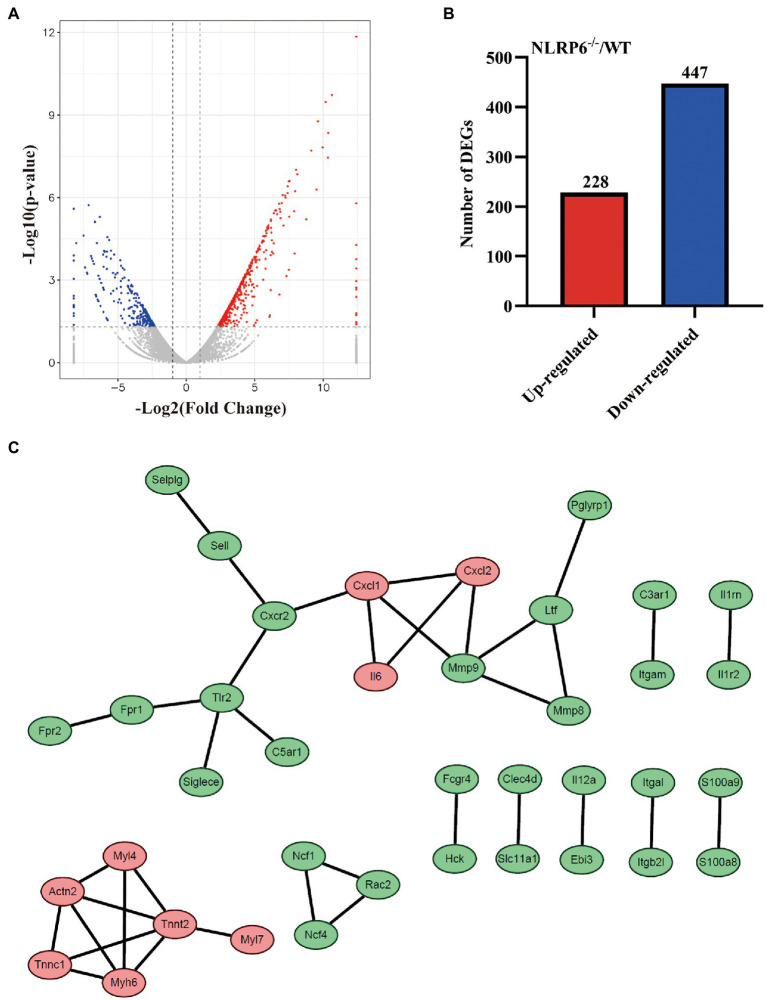
DEGs identification of lung tissue from *S. pneumoniae*-infected WT and NLRP6^−/−^ mice. WT and NLRP6^−/−^ mice were intranasally infected with *S. pneumoniae* (5 × 10^7^ CFU) for 24 h and then lung tissues were collected for transcriptome analysis. **(A)** Volcano plot of differentially expressed genes (DEGs). The two vertical dotted lines are the twofold expression difference threshold, the horizontal dotted line represent value of *p* = 0.05. **(B)** The up/down-regulated DEGs of WT and NLRP6^−/−^. **(C)** Protein–protein interaction (PPI) network of the top 200 DEGs with the most significant difference in value of *p* (score > 0.95). The red circles represent the upregulated DEGs, the green circles represent the downregulated DEGs.

To find the critical factors that drive NLRP6 to exert negative function *in vivo* against *S. pneumoniae* infection, the differentially expressed genes (DEGs) in lung tissues of WT and NLRP6^−/−^ mice infected with *S. pneumoniae* were compared using transcriptome sequencing (RNAseq). RNAseq analysis showed that a total of 675 genes were significantly different between WT and NLRP6^−/−^ mice lung tissue infected with *S. pneumoniae* (*p* < 0.05). Of these DEGs, 228 genes were significantly up-regulated and 447 genes were significantly down-regulated (Figures 2A,B). The top 20 upregulated DEGs mainly contain *Clca1*, *Myh6*, *Myl7*, *Cxcl1*, and *Tspan18* (Supplementary Table 1) and downregulated DEGs mainly include *Mrgpra2b*, *Asprv1*, *Mmp8*, *Ubd*, and *C3ar1* (Supplementary Table 2). To identify the most significant clusters of the top 200 DEGs with the most significant difference in value of *p*, protein–protein interaction (PPI) network of DEGs was constructed by STRING and visualized by *Cytoscape*. One hub gene module consisted of 15 genes, including *Cxcl1*, *Cxcl2*, *Il6*, *Mmp8*, *Mmp9*, *Ltf*, *Pglyrp1*, *Cxcr2*, *Sell*, *Selplg*, *Tlr2*, *C5ar1*, *Siglece*, *Fpr1*, *Fpr2* (Figure 2C).

### GO and KEGG Analysis of DEGs

**Figure 3:**
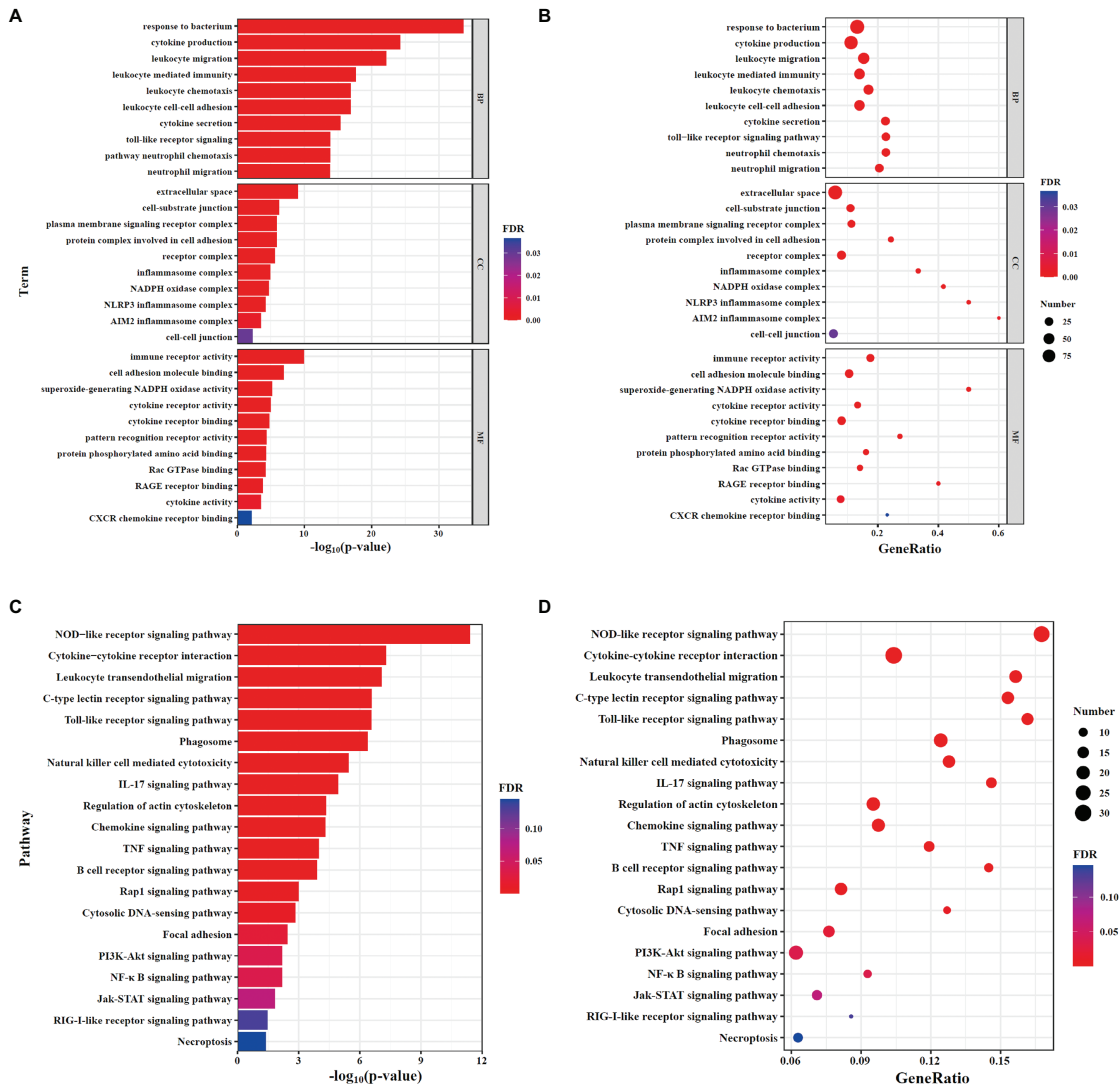
GO and KEGG analysis of DEGs. **(A,B)** The gene ontology (GO) enrichment analysis of DEGs. **(A)** Barplot; **(B)** Dotplot. **(C,D)** Kyoto encyclopedia of genes and genomes (KEGG) pathway enrichment analysis of DEGs. **(C)** Barplot; **(D)** Dotplot. The x-axis shows the -log10(value of p; **A,C**) and gene ratio **(B,D)** of each term, and y-axis shows the GO annotation terms **(A,B)** and the KEGG pathway terms **(C,D)**.

To analyze the functional level of DEGs, gene ontology (GO) and Kyoto encyclopedia of genes and genomes (KEGG) analysis was performed. Results of GO analysis showed that changes in biological process (BP) of DEGs were enriched in cytokine production, leukocyte migration, and leukocyte chemotaxis. The cellular component (CC) analysis showed that cell–substrate junction, inflammasome complex and NADPH oxidase complex were significantly altered. DEGs linked with molecular function (MF) were enriched in immune receptor activity, cell adhesion molecule binding, and superoxide-generating NADPH oxidase activity (Figures 3A,B). The top 10 GO terms in BP, CC, and MF with the significant enrichment were shown in Supplementary Figures 1A,B including defense response, cell surface, and protein binding. Next, KEGG enrichment analysis was performed to further determine the signaling pathways associated with NLRP6 function during *S. pneumoniae* infection. The results showed that the signaling pathways including NOD-like receptor signaling pathway, leukocyte transendothelial migration, chemokine signaling pathway, and TNF signaling pathway were significantly different between WT and NLRP6^−/−^ lung tissues (Figures 3C,D). The top 20 pathways with the most significant differences in KEGG analysis were shown in Supplementary Figures 2A,B. These results suggest that NLRP6 modulates the migration and recruitment of leukocytes.

### NLRP6 Deficiency Promotes the Recruitment of Neutrophils and Macrophages by Regulating Chemokine Expression

**Figure 4:**
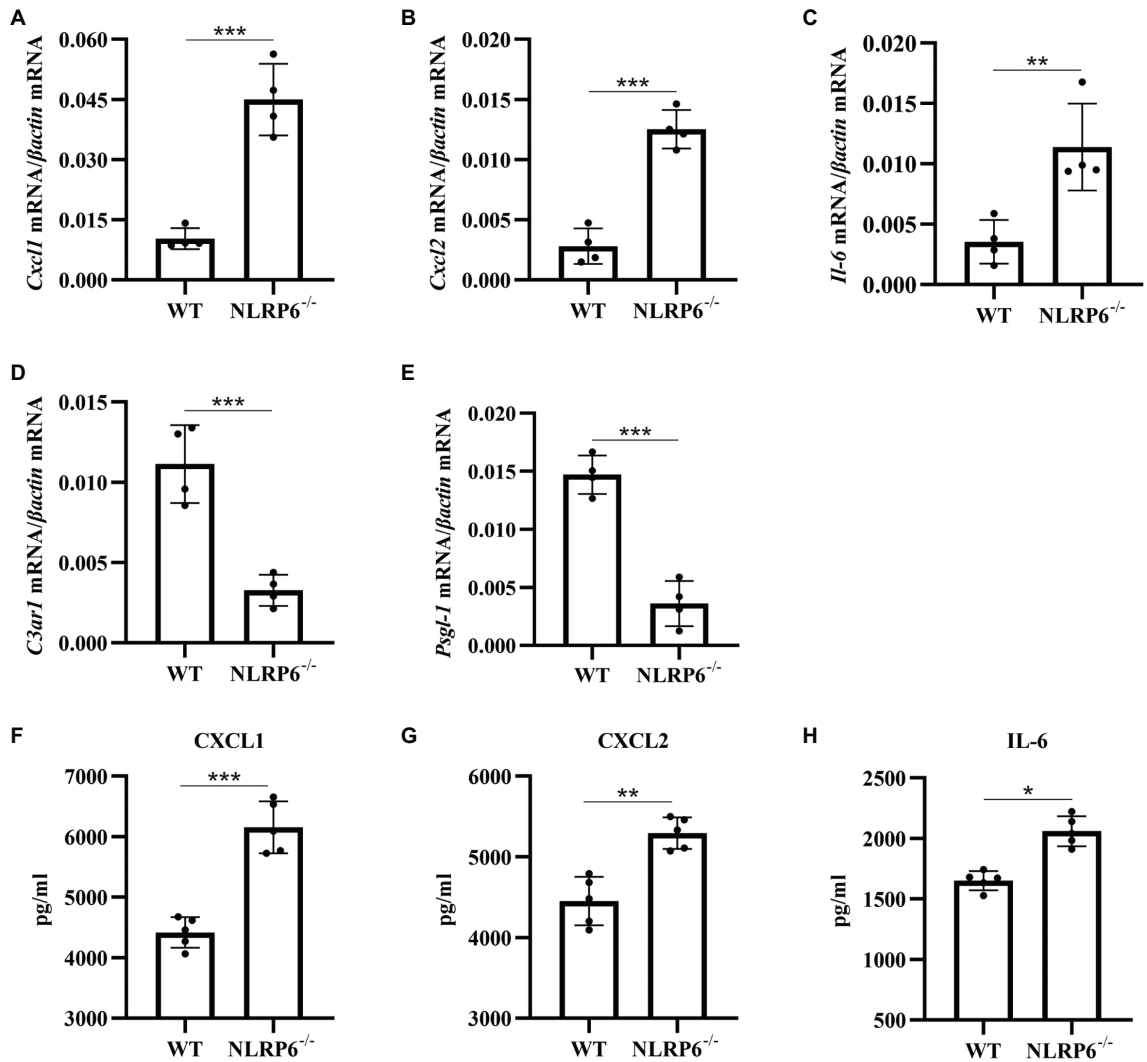
NLRP6 deficiency promotes the recruitment of neutrophils and macrophages by regulating chemokine expression. WT and NLRP6^−/−^ mice were intranasally infected with *S. pneumoniae* (5 × 10^7^ CFU) for 24 h and then lung tissues were collected. Transcript levels of *Cxcl1* **(A)**, *Cxcl2* **(B)**, *Il-6* **(C)**, *C3ar1* **(D)**, *Psgl-1* **(E)** normalized with *β-actin* were quantified (*n* = 4/group). The levels of CXCL1 **(F)**, CXCL2 **(G)**, and IL-6 **(H)** were measured by ELISA (*n* = 5/group). Statistical significance was shown as *p < 0.05, **p < 0.01, and ***p < 0.001.

Our previous study has reported that the number of neutrophils and macrophages were significantly increased in *S. pneumoniae*-infected NLRP6^−/−^ mice (Xu et al., 2021). To further investigate the mechanism by which NLRP6 regulates leukocyte recruitment and migration-related pathways, we examined gene expression related to migration and recruitment of macrophages and neutrophils in the lung of WT and NLRP6^−/−^ mice after *S. pneumoniae* infection. The results showed that mRNA expression of CXCL1/2 and IL-6 were significantly upregulated in *S. pneumoniae*-infected NLRP6^−/−^ lung tissue compared with WT mice (Figures 4A–C). However, C3aR1 has been reported as a negative regulator to inhibit the expression of CXCL2 (Brennan et al., 2019) and our results showed that mRNA expression of C3aR1 was significantly downregulated in NLRP6^−/−^ lung tissue during *S. pneumoniae* infection (Figure 4D). Furthermore, PSGL-1 has shown to inhibit neutrophil migration (Levesque et al., 1999) and our results showed that its gene expression was also significantly downregulated in NLRP6^−/−^ lung tissue (Figure 4E). Similarly, protein expression of CXCL1, CXCL2 and IL-6 was also significantly increased in *S. pneumoniae*-infected NLRP6^−/−^ mice (Figures 4F-H). Together, these results suggest that NLRP6 plays a negative role on regulating migration and recruitment of immune cells during *S. pneumoniae* infection.

### NLRP6 Deficiency Enhances Neutrophil Intracellular Bactericidal Capacity and Macrophage Viability

**Figure 5:**
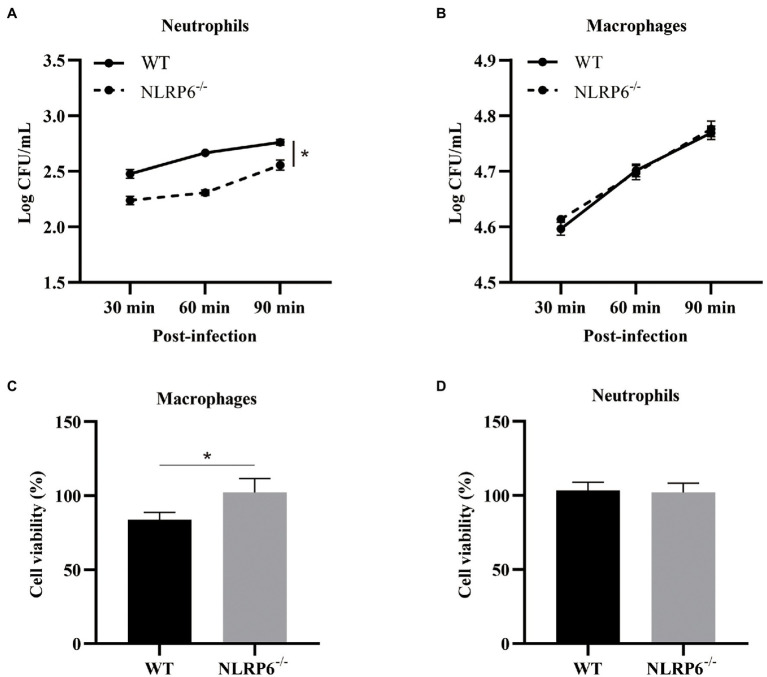
NLRP6 deficiency enhances neutrophil intracellular bactericidal capacity and macrophage viability. Neutrophils and macrophages from WT and NLRP6^−/−^ mice. Neutrophils and macrophages were infected with *S. pneumonia*e (MOI = 10) and the intracellular bactericidal capacity of neutrophils **(A)** and macrophages **(B)** was measured at 30, 60, and 90 min by estimating intracellular CFU using 6 wells/group. The cell viability of macrophages **(C)** at 24 h and the cell viability of neutrophils **(D)** at 12 h after infection with *S. pneumoniae* (MOI = 1) were determined by WST-1 assay (*n* = 3). Statistical significance was shown as *p < 0.05.

To further investigate whether NLRP6 regulates the function of macrophages and neutrophils, their intracellular bactericidal capacities were determined. The results showed that intracellular bactericidal ability of neutrophils was enhanced and associated with reduced number of intracellular bacteria in the absence of NLRP6 compared to WT neutrophils (Figure 5A). However, NLRP6^−/−^ macrophages did not show similar killing ability as neutrophils (Figure 5B). Next, macrophages and neutrophils viability were determined after *S. pneumoniae* infection and the results showed that WT macrophage viability was attenuated compared to NLRP6^−/−^ macrophages (Figure 5C) while *S. pneumoniae* infection did not affect WT and NLRP6^−/−^ neutrophil viability (Figure 5D). These results indicate that NLRP6 inhibits neutrophil intracellular bactericidal ability and reduces macrophage viability against *S. pneumoniae*.

### NLRP6 Inhibits Formation of NETs of Neutrophils

**Figure 6:**
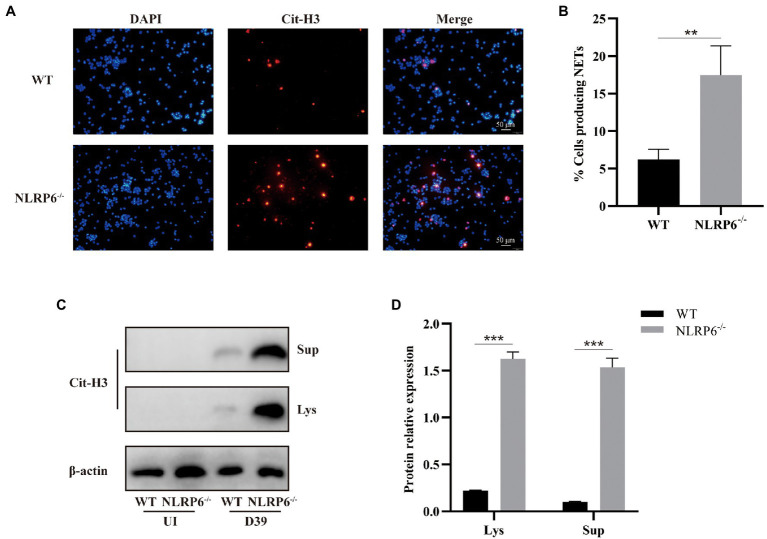
NLRP6 inhibits formation of NETs of neutrophils. Neutrophils from WT and NLRP6^−/−^ were infected with *S. pneumoniae* at an MOI of 1 for 4 h. Immunofluorescent staining was to detect the expression of citrullinated-H3 (Cit-H3) which is the important component of NETs (*n* = 3). The representative images of Cit-H3 are shown **(A)**. Quantification of neutrophils with NETs **(B)**. The culture supernatants and cell lysates were analyzed by western blot to detect expression of Cit-H3 (**C**; *n* = 3). Ratio of Cit-H3 levels against β-actin levels was quantified **(D)**. Statistical significance was shown as **p < 0.01 and ***p < 0.001.

Neutrophils considered as the first defense line against microbial infection have different weapons to eradicate infectious agents (Tourneur and Witko-Sarsat, 2019). One of the mechanisms by which neutrophils play a defensive role is to capture and eliminate pathogens by releasing NETs (Brinkmann et al., 2004). To determine whether NLRP6 enhances neutrophil bactericidal capacity by regulating the release of NETs of neutrophils, the expression of citrullinated histone 3 (Cit-H3) was detected during *S. pneumoniae* infection. Immunofluorescent staining showed that the expression of Cit-H3 was significantly higher in NLRP6^−/−^ neutrophils than in WT neutrophils after *S. pneumoniae* infection (Figures 6A,B), and similar results were also observed in western blot assay (Figures 6C,D). These results suggest that NLRP6 negatively regulates NETs formation during *S. pneumoniae* infection, leading to the damage of neutrophil intracellular killing activity.

## Discussion

*Streptococcus pneumoniae* is the leading cause of bacterial pneumonia and sepsis worldwide, especially in developing countries (Dong et al., 2020). So far, the treatment of bacterial pulmonary infection mainly relies on antibiotics, but increased antibiotics resistance has been a great challenge to treat bacterial infection. Therefore, the novel therapeutics are urgently needed. Host innate response is critical to defend invade pathogens and thus the detailed understanding of host signaling events might contribute to the development of new potential therapeutic interventions. Inflammasomes play a critical role in the host innate immunity defense against microbial infection (Hara et al., 2018; Paudel et al., 2019b; Venuprasad and Theiss, 2021). For example, NLRP3 inflammasome has been reported to contribute to the clearance of invading pathogens (Jin et al., 2017). NLRP6 inflammasome is also found to be required in the host defense against some pathogens, such as *C. rodentium* and *K. pneumoniae* (Wlodarska et al., 2014; Birchenough et al., 2016; Cai et al., 2021). However, the contradictory role of NLRP6 has been reported during *L. monocytogenes*, *S. typhimurium*, *E. coli* and *S. aureus* infection (Anand et al., 2012; Ghimire et al., 2018). Our previous study has shown that NLRP6^−/−^ mice has low mortality against *S. pneumoniae* infection, but the mechanism by which NLRP6 negatively modulate the host signaling is still unclear.

Tight junction protein is an important component that maintains the selective passage of paracellular substances and enhances the function of cell barriers (Otani and Furuse, 2020). A variety of pathogenic bacteria cause paracellular barrier dysfunction by reducing the expression of tight junction proteins such *Bacillus anthracis* (Ebrahimi et al., 2009), *Neisseria meningitidis* (Schubert-Unkmeir et al., 2010), and *S. aureus* (McLoughlin et al., 2017). Our current study found that NLRP6 deficiency can maintain the expression of tight junction protein occludin in the lung during *S. pneumoniae* infection, indicating that NLRP6 plays a detrimental role in the epithelial barrier against bacterial infection.

Neutrophils recruited to infection site is critical for the clearance of invading pathogens and they exhibit function through phagocytosis, the release of antimicrobial compounds and the formation of NETs (Papayannopoulos, 2018). It has been reported that NLPR6^−/−^ mice displayed increased neutrophils and macrophages in the alveoli in *S. aureus-*induced pulmonary infection (Ghimire et al., 2018). Our previous study confirmed that NLRP6 negatively modulated the recruitment of macrophages and neutrophils (Xu et al., 2021). In agreement with these findings, our transcriptome analysis indicates that NLRP6 was involved in regulating the recruitment of leukocytes during *S. pneumoniae* infection. In contrast, Cai et al. have shown that NLRP6 deletion impairs neutrophil recruitment during *K. pneumoniae* infection (Cai et al., 2021). It is likely that *K. pneumoniae* infection failed to induce robust inflammatory or pathological response, thus NLRP6 acting as a positive regulator of host defense. It is well known that cytokines/chemokines drive the recruitment of leukocytes (Paudel et al., 2019a). Cai et al. reported that recombinant CXCL1 rescue the recruitment of neutrophils in *K. pneumoniae*-infected NLRP6^−/−^ mice, indicating the critical role of CXCL1 in the recruitment of neutrophils. Our current study showed that NLRP6 deletion significantly upregulated the expression of cytokine and chemokines including CXCL1, CXCL2 and IL-6 that drive the recruitment of leukocytes during *S. pneumoniae* infection. In contrast, our previous study showed a decrease of IL-6 protein in the BALF of NLRP6^−/−^ mice during *S. pneumoniae* infection (Xu et al., 2021). This discrepancy of IL-6 protein expression in these two studies may be the results of different infection time and different sample collections. It could be possible that IL-6 expression is not static and its expression changes with different infection times. Recent reports demonstrate that PSGL-1 reduces the number of neutrophils and macrophages in the blood during *S. pneumoniae* infection (Ramos-Sevillano et al., 2016). Furthermore, Brennan et al. have shown that C3aR1 acts as a physiological antagonist of neutrophil chemotactic signaling and inhibits CXCL2-driven neutrophil mobilization following trauma (Brennan et al., 2019). Our study showed that NLRP6^−/−^ mice had downregulated gene expression of PSGL-1 and C3aR1 that negatively drive neutrophil chemotactic signaling during *S. pneumoniae* infection. These studies indicate that NLRP6 inhibits the recruitment of neutrophils through attenuating the activation of chemotactic signaling pathway.

Neutrophils and macrophages as the main bactericidal cells in the initial infection play a critical role in the clearance of pathogens (Ramos-Sevillano et al., 2016). Especially, neutrophils as the fastest responder are recruited to infected sites and their bactericidal capacity has a positive effect on protecting the host against invade pathogens (Capucetti et al., 2020). It has been reported that NLRP6 can modulate intracellular bactericidal ability of neutrophils (Ghimire et al., 2018). Ghimire et al. found that NLRP6 deletion neutrophils displayed stronger intracellular bacterial killing against *S. aureus* compared to WT neutrophils, while macrophage intracellular bacterial killing was not changed (Ghimire et al., 2018). Similarly, our study also indicates that NLRP6 negatively regulate intracellular bacterial killing of neutrophils against *S. pneumoniae* infection. Additionally, NLRP6 deficiency can maintain macrophage viability during *S. pneumoniae* infection. NLRP6 also negatively regulated other cell type function such as hematopoietic and non-hematopoietic cells with increased susceptibility to *Listeria* and *Salmonella* infections (Anand et al., 2012). These studies demonstrate the detrimental role of NLRP6 signaling in the host defense. In contrast with those bacterial infections as mentioned above, NLRP6 was found to be a central regulator of neutrophil recruitment and function in response to *K. pneumoniae* infection (Cai et al., 2021). Furthermore, some researches have shown that NLRP6 has a protective role in intestine health through regulating gut microbiota composition (Wlodarska et al., 2014). Although the exact mechanism by which NLRP6 plays a dual role in the host is still unclear, it is clear from our investigation that NLRP6 is detrimental to neutrophils intracellular killing function.

The formation of NETs is the central role of neutrophils in killing pathogens. Different cellular signaling is involved in the formation of NETs. Dong et al. found that TLR4 was required for the formation of NETs of neutrophils by regulating ROS and autophagy during *S. pneumoniae* infection (Dong et al., 2021). Cai et al. found that NLRP6^−/−^ neutrophils displayed decreased NETs formation and NET-mediated bacterial killing during *K. pneumoniae* infection (Cai et al., 2021). In contrast, here we show that NLRP6 deletion enhanced NETs formation. It has been reported that the formation of NETs is dependent on ROS generation by NADPH oxidase complex (Rosazza et al., 2021). Recent studies have demonstrated that NLRP6 deficiency increases ROS expression upon *S. aureus* infection (Ghimire et al., 2018), suggesting NLRP6-mediated NETs formation might be regulated by ROS production.

In summary, NLRP6 played a detrimental role in the host defense against *S. pneumoniae* by reducing neutrophil recruitment and function through the decreased production of chemokines. Furthermore, NLRP6 negatively regulated the intracellular bactericidal capacity of neutrophils and the formation of NETs.

## Data Availability Statement

The raw Illumina reads of RNA-seq are deposited in the NCBI Sequence Read Archive repository, accession number PRJNA837015.

## Ethics Statement

The animal study was reviewed and approved by Institutional Animal Care and Use Committee of Southwest University.

## Author Contributions

QT, DX, KJ, and XC performed experiments. CY, SX, and D-LH helped to analyze the data. RF and LP supervised the study and designed the experiments. QT, LP, D-LH, and RF drafted the manuscript. All authors have read and agreed to the published version of the manuscript.

## Funding

This study was supported by the National Key Research and Development Program of China (2021YFD1800800), National Natural Science Foundation of China (32172850 and 32102684), the Chongqing Science & Technology Commission (cstc2021jcyj-msxmX0504 and cstc2021jcyj-msxm2941), Chongqing Pig Industry Technology System (20211105), and the Foundation for Innovation Research Groups in Chongqing Universities (CXQT20004).

## Conflict of Interest

The authors declare that the research was conducted in the absence of any commercial or financial relationships that could be construed as a potential conflict of interest.

## Publisher’s Note

All claims expressed in this article are solely those of the authors and do not necessarily represent those of their affiliated organizations, or those of the publisher, the editors and the reviewers. Any product that may be evaluated in this article, or claim that may be made by its manufacturer, is not guaranteed or endorsed by the publisher.

## Supplementary Material

Supplementary Figure 1 GO analysis of DEGs in *Streptococcus pneumoniae*-infected lungs of WT and NLRP6-/- mice. The top 10 GO terms in BP, CC and MF with the significant enrichment. **(A)** Barplot; **(B)** Dotplot. The x-axis shows the log10(p-value; **A**) and gene ratio **(B)** of each term, and y-axis shows the GO annotation terms.

Supplementary Figure 2 KEGG analysis of DEGs in *S. pneumoniae*-infected lungs of WT and NLRP6-/- mice. The top 20 pathways with the most significant differences in KEGG analysis. **(A)** Barplot; **(B)** Dotplot. The x-axis shows the log10(p-value; **A**) and gene ratio **(B)** of each term, and y-axis shows the KEGG pathway terms.
