# Supplementary material for: NLRP6 Serves as a Negative Regulator of Neutrophil Recruitment and Function During Streptococcus pneumoniae Infection
Source: Front Microbiol. 2022 May 25;13:898559. doi: 10.3389/fmicb.2022.898559 (PMC9174927; doi:10.3389/fmicb.2022.898559)
Supplement: Supplementary file 1 [file Table_1.DOCX]

**Table1.** The top 20 upregulated DEGs between NLRP6^-/-^ and WT group

| Gene Name | baseMean | log_2_(Fold Change) | p-value | adj. p-value |
| --- | --- | --- | --- | --- |
| *Clca1* | 1334.240856 | 6.863306032 | 2.88437E-15 | 4.93631E-11 |
| *Myh6* | 1693.238036 | 3.821013278 | 7.06453E-08 | 0.000109911 |
| *Myl7* | 366.6762641 | 4.87433481 | 9.0066E-08 | 0.000118568 |
| *Cxcl1* | 5507.823046 | 3.096737708 | 2.48535E-06 | 0.001701373 |
| *Tspan18* | 1051.074631 | 3.349227018 | 2.72171E-06 | 0.001745925 |
| *Mmp12* | 902.2923777 | 3.203796578 | 8.03925E-06 | 0.00359225 |
| *Scel* | 808.2235979 | 3.241992902 | 8.18615E-06 | 0.00359225 |
| *Hpgd* | 8738.797667 | 2.761523541 | 1.79716E-05 | 0.006938331 |
| *Cyp26b1* | 867.7364994 | 3.058191256 | 1.86492E-05 | 0.006938331 |
| *Lrtm2* | 906.1319198 | 2.821997027 | 5.90278E-05 | 0.018318105 |
| *Sftpc* | 155620.4781 | 2.477100972 | 8.49443E-05 | 0.02463961 |
| *Atp6v0d2* | 1422.550323 | 2.526454967 | 0.000163839 | 0.036894068 |
| *Myl4* | 389.7135163 | 2.9622419 | 0.000168448 | 0.037439156 |
| *Cxcl2* | 1899.613421 | 2.385341154 | 0.000279661 | 0.056977511 |
| *Cavin2* | 4274.370168 | 2.25423753 | 0.000404442 | 0.073634291 |
| *Tnnt2* | 1251.690703 | 2.360231918 | 0.000428523 | 0.076393092 |
| *Col4a4* | 2381.475946 | 2.275931454 | 0.000442074 | 0.077200514 |
| *Edn3* | 127.6647726 | 3.937150919 | 0.000453016 | 0.078312317 |
| *Ptgs2* | 2235.573349 | 2.272947541 | 0.000462455 | 0.07854219 |
| *Tmem44* | 643.1232905 | 2.462530846 | 0.000550447 | 0.086094445 |
