# Supplementary material for: NLRP6 Serves as a Negative Regulator of Neutrophil Recruitment and Function During Streptococcus pneumoniae Infection
Source: Front Microbiol. 2022 May 25;13:898559. doi: 10.3389/fmicb.2022.898559 (PMC9174927; doi:10.3389/fmicb.2022.898559)
Supplement: Supplementary file 2 [file Table_2.DOCX]

**Table2.** The top 20 downregulated DEGs between NLRP6^-/-^ and WT group

| Gene Name | baseMean | log_2_(Fold Change) | p-value | adj. p-value |
| --- | --- | --- | --- | --- |
| *Mrgpra2b* | 312.0048888 | -5.614908492 | 1.39465E-10 | 6.80014E-07 |
| *Asprv1* | 6286.977794 | -4.437186338 | 1.57533E-10 | 6.80014E-07 |
| *Mmp8* | 7300.314208 | -4.428579362 | 1.58937E-10 | 6.80014E-07 |
| *Ubd* | 1159.626132 | -4.548795251 | 3.76201E-10 | 1.28766E-06 |
| *C3ar1* | 1967.13342 | -4.032744685 | 6.11578E-09 | 1.74442E-05 |
| *Cstdc4* | 1474.014239 | -3.997122343 | 1.07012E-08 | 2.61629E-05 |
| *Stfa3* | 580.0972321 | -4.06821563 | 3.8656E-08 | 8.26949E-05 |
| *Psgl1* | 3842.403927 | -3.649209456 | 5.58173E-08 | 9.55257E-05 |
| *Stfa2* | 897.0718429 | -3.802501446 | 7.85356E-08 | 0.000112005 |
| *Ifi208* | 1529.581236 | -3.644348903 | 1.08155E-07 | 0.000132212 |
| *Retnlg* | 5191.031154 | -3.425417631 | 2.33521E-07 | 0.000266432 |
| *Il1f9* | 1433.847841 | -3.478255935 | 3.41085E-07 | 0.000364833 |
| *Marcksl1* | 4837.425044 | -3.317237749 | 4.959E-07 | 0.000499225 |
| *Stfa2l1* | 1238.055081 | -3.430144143 | 5.39453E-07 | 0.0005129 |
| *Slfn4* | 42384.01629 | -3.242727071 | 6.34528E-07 | 0.000571543 |
| *Mgam* | 220.3567515 | -4.118131517 | 9.14068E-07 | 0.000782168 |
| *Fcer2a* | 239.9460153 | -3.969464136 | 1.26343E-06 | 0.001029632 |
| *Cstdc5* | 92.55558045 | -5.561075013 | 1.32572E-06 | 0.001031291 |
| *S100a8* | 18406.35941 | -3.069167086 | 2.11952E-06 | 0.00155798 |
| *S100a9* | 34054.91911 | -3.059839972 | 2.18485E-06 | 0.00155798 |
