# Supplementary figures and images for: NLRP6 Serves as a Negative Regulator of Neutrophil Recruitment and Function During Streptococcus pneumoniae Infection
Source: Front Microbiol. 2022 May 25;13:898559. doi: 10.3389/fmicb.2022.898559 (PMC9174927; doi:10.3389/fmicb.2022.898559)

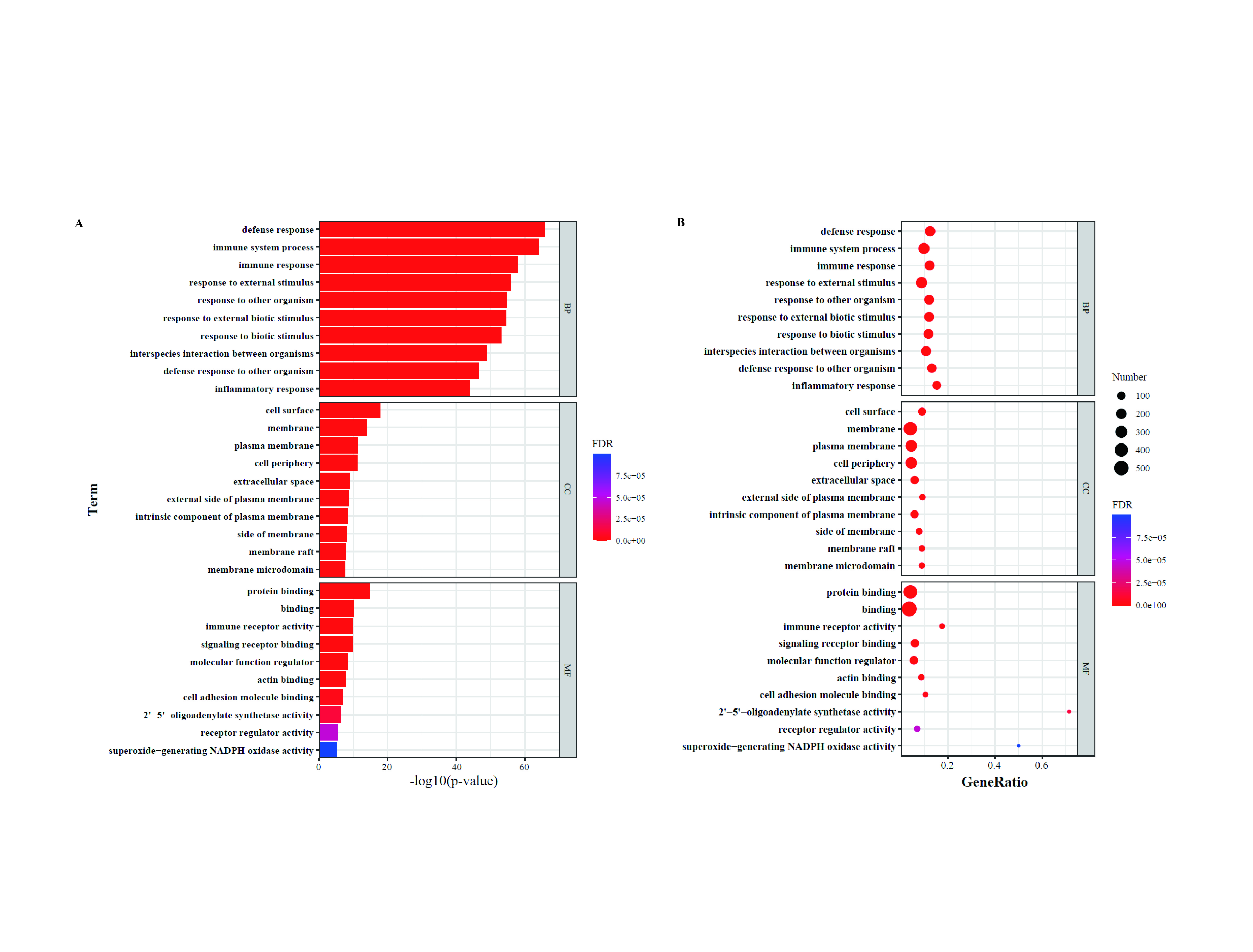

Supplement: Supplementary Figure 1 — GO analysis of DEGs in Streptococcus pneumoniae-infected lungs of WT and NLRP6-/- mice. The top 10 GO terms in BP, CC and MF with the significant enrichment. (A) Barplot; (B) Dotplot. The x-axis shows the log10(p-value; A) and gene ratio (B) of each term, and y-axis shows the GO annotation terms. [file Image_1.JPEG]

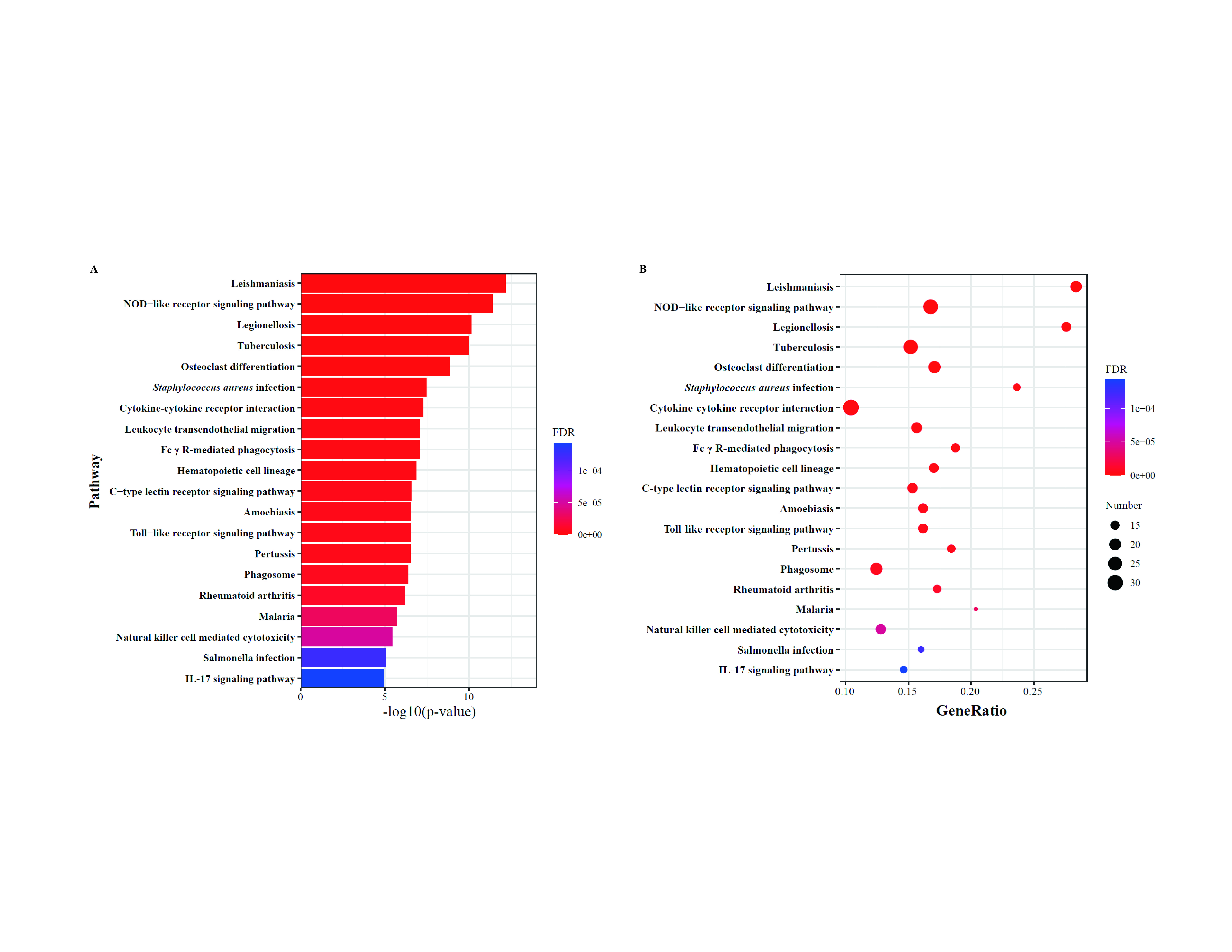

Supplement: Supplementary Figure 2 — KEGG analysis of DEGs in S. pneumoniae-infected lungs of WT and NLRP6-/- mice. The top 20 pathways with the most significant differences in KEGG analysis. (A) Barplot; (B) Dotplot. The x-axis shows the log10(p-value; A) and gene ratio (B) of each term, and y-axis shows the KEGG pathway terms. [file Image_2.JPEG]
